# Hermaphrodism, from a Medico-Legal Point of View

**Published:** 1875-11

**Authors:** 


### Translations.

### HERMAPHRODISM,

FROM A MEDICO-LEGAL POINT OF VIEW.

*Translated from the French of Basile Poppesco,*

By E. WARREN SAWYER, M.D.,

Lecturer on Obstetrics, Rush Medical College, Chicago.

(Continued from page 792.)

We have undertaken, at some length, to state these ingenious explanations, without being understood as adopting purely speculative theories.

Other authors have given another explanation of hermaphrodism; I will limit myself to the simple mention of it; among others, I will cite the names of Is. Geoffroy Saint-Hilaire, Dutrochet and Marc. According to these authors, who assume as the foundation of the fundamental law of the development of the sexual apparatus, that the most complete analogy exists between the male and female organs, to that degree, even, that in the early moments of their formation, it is not easy to distinguish them ; and that later, each part of the male apparatus has its analogue in the female apparatus, and reciprocally ; according to these authors, I say, it is not till the end of a certain period of intra-uterine life, that these elements, primitively neuter, will become male or female, according to the reciprocal impulses transmitted from the male ovule to the female ovule. This is the theory that we are enabled to call the *neutrality of the sexes* during intra-uterine life, which will explain why, under certain given circumstances, the transmitted impulse is insufficient to bring about a normal development, and, consequently, the entire series of analogous transformations in the different divisions of the generative organs.

Another school supposes the following principle ; and admits a *normal physiological bisexuality.* According to the leaders of this school, every fœtus possesses the two sexual apparatuses capable of taking on a complete development, but usually, and especially according to the law of Serres, known under that of the equilibrium of the organs, one of these apparatuses should normally atrophy ; whence results, in consequence, a complete male or female sexual apparatus. But, for another reason, still ignored : the laws of nature suffer a deviation, in order that there may be any arrest of development, the homogeneity is destroyed, and the consequence will be the formation of the generative apparatus, after an extravagant and singular fashion, exhibiting the union of the male and female organs. This is an extremely ingenious theory, but it cannot resist one serious criticism founded upon the recent results of modern embryology, and especially of histology. Nevertheless, it is apparent how easy it will be to explain, by this means, the positive, but exceptional facts of bisexual hermaphrodism, such as we have detailed in our observations V and VI.

In conclusion, I will call attention to the complete independence, the slight relations, so to speak, which exist, at least during the first months of intra-uterine life, between the fundamental organs of generation and the accessory organs, in both sexes ; "whence results," says Professor Tardieu, "upon the one hand, the possibility of an incomplete evolution, which will retain certain parts in a primitive state, whilst other parts will develop normally ; and upon the other hand, the confusion of the characteristics of the sex, which will be female on one side and male on the other."

### APPLICATION OF THE PRECEDING DATA TO LEGAL MEDICINE.

Several medico-legal questions of great gravity belong to the vices of conformation of the sexual organs. Sometimes the individuals, who have had a false social rank from the time of birth, are condemned to a series of moral disturbances, before being able to recover their title, and to receive their common right. Sometimes a marriage, contracted under the conditions of identity of sex, has been declared null. As M. Tardieu has said, "from all the points of view, moral, physiological and social, these facts are of a nature to interest profoundly the philosopher and the physician."

The essential object of marriage being the union of the sexes, it results from this, that the difference of sex is the essential condition ; article 144 of the Civil Code supposes this condition, otherwise a marriage does not exist. Also, when by a concourse of extraordinary circumstances, of which numerous examples are found in our old law, and even in the present, (decree of the Parliament, 1765, decree of the court of Trêves, 1808), when a union so monstrous is found, there is not a marriage, but a *representation of marriage.*

If the apparent silence of the Code, on the variety of causes of impotence, has been the cause of the division which still reigns among the jurists, it seems difficult to us, on the other hand, to include hermaphrodism among the causes of *impotence*, as M. Legrand du Saulle has said, when that impotence, even the most notorious, is not a cause of opposition to the marriage; on the contrary, article 180 of the Civil Code sufficiently instructs the physician and the judge concerning the vices of conformation, which constitute hermaphrodism. In fact, such are the expressions of the Code, when there is a *fault in the person*, the marriage cannot be challenged by another than one of the two espoused who has been led into error.

The law not having made mention of the word barrenness, we should give to the word impotence a legal significance quite different, and much more comprehensive, than when the same word is employed in a strictly medical sense. Always under the circumstances, when the impotence has been well marked, the courts have simply compared the fact to the cases in which there has been a fault in the person, such as enables us to pronounce upon the invalidity of the marriage.

Not wishing to further enlarge upon the causes of impotence, or upon the manner of proceeding in order to discover the truth and to render it visible and palpable, so to speak, to the eyes of the court, especially when concerned with questions so delicate, we will now examine the different questions which can be submitted to the medical expert, constantly recalling the fact that, in the majority of instances, there is a fault in the person ; and that it is this fact which establishes either the invalidity of the marriage, or demands simply the revision of the social state, in consequence of a vicious conformation of the genital parts, which has given rise to the doubt or the fault.

Consider the first case, that which is presented most commonly, under circumstances that are generally well indicated. Marc, and Orfila, in their treatises on legal medicine, have, among others, reported the history of Marie Marguerite, who, wishing to marry, was visited by a surgeon on account of the absolute absence of menstruation; and because of which, the tribunal of Dreux had directed her to assume male attire. Briand and Chaudé also speak of this individual who, christened and educated as a girl, one day demanded the privilege of marrying a woman whose pregnancy was due to his efforts.

The same authors still further express themselves: "Marriage cannot be otherwise contracted than between two persons of different sex; if it is to be supposed that a marriage might have been contracted between a woman and an individual who, up to that time, had been regarded as belonging to the male sex, but who really was a woman, as she was, will any one dare to assume that such a marriage is valid? No; without doubt such a union ought evidently to be annulled, because of error. Let one suppose, on the contrary, an individual in whom a freak of nature had given birth to a rudimentary virile organ, is there not equally in this case an error in the person? In both cases, the justice of the decision is the same, *ubi eadem ratio, idem jus.* It is necessary to annul such a marriage." Moreover, it was thus that the case was decided which came at first before the tribunal at Alais, in 1869; I desire to recall, in a few lines, the interesting particulars in this case. M. D. was married in 1866 to a person inscribed on the register of civil state under the name and surname of Justine A. Z., then twenty-five years of age. In 1869, only, he decided to demand the nullification of his marriage, for which he alleged the following motives: according to his own observations and those of a midwife, this pretended married woman did not possess a single organ which characterizes the female sex; she had neither breasts, ovaries, uterus nor vagina; her pelvis was conformed to that of the male rather than to that of the female; moreover, though she was twenty-seven years of age, she had never had either menses or periodical lumbar and abdominal pains. After a first decision had been reversed by the Imperial Court of Nîmes, which among other considerations gave the following: "While the law has not included impotence with the causes for nullifying a marriage, it follows that every demand, tending to prove that one of the espoused is found in that state, should be rejected," the cause was then submitted to the supreme court. It was then that M. Tardieu was appealed to for his opinion.

"The intervention of the physician, indispensable in such cases, is perfectly defined in its object, and should be, at the same time, perfectly distinct in its results. The question to decide is expressed in these very simple terms: the person espoused as a woman, is she a malformed woman, impotent and unfit for sexual union? In this case, there is not a cause for divorce, in the restricted sense the law has fixed. Is he a malformed man offering the deceitful appearances of the female sex? In this case, the marriage has not even existed, and is radically wrong. A medico-legal expert is, without doubt, necessary and indispensable, for the solution of the double question that I have just indicated."

Relying upon a very incomplete certificate, given by Dr. C—, M. Tardieu, taking as a foundation for his scientific argument, the general conformation, the narrowness of the pelvis, the negative development of the breasts, the absence of the vagina, but especially the absolute failure, not only of the original menstrual flow, but even, at that time, of the periodical flux, accompanied by lumbar or abdominal pains, with swelling of the breasts, arrived at the conclusion, that this person, not possessing in reality a single organ essential to the constitution of the female sex, was a man, but a malformed man, presenting the most common type of hermaphrodism.

In adding to his report, M. Tardieu recalled the fact that, from a medico-legal point of view, sexual indifference, or absolute neutrality does not exist; and Briand and Chaudé themselves, after having admitted theoretically this variety of hermaphrodism, say that, from a medico-legal point of view, "these individuals should be regarded as being of the masculine sex; because, in them the female genital parts cannot be discovered; and the absence of virility depends only then upon the want, or the atrophy of the testicles."

The case was only decided in 1873, and the court annulled the marriage inscribed on the registers of the civil state of the commonwealth of Alais.

One can resume all which has preceded, in saying that, if the impotence or the infecundity of the espoused cannot be admitted as a cause for nullification of marriage, it is not the same for identity of sex, which requires most often a declaration of fault in the person, and consequently establishes the nullity of the marriage. This case is certainly the most difficult which can be submitted to the examination of the medical expert.

ERRORS IN THE INSCRIPTION OF THE SEX IN THE CIVIL STATE.

In addition to the foregoing facts, there remains to be considered the *errors in the inscription of the sex in the civil state.* These facts have a capital importance; because, if an error is committed, there will result a series of disastrous consequences which I have just indicated in the preceding pages.

It will be remembered that the greater part of the vices of conformation are the result of an arrest in development, the subject of which, in the immense majority of cases, is an individual belonging to the male sex; and that the malformations of the sexual organs of the female, are much more easy to recognize, especially on account of the multiple and varied manifestations the ovaries are subject to, by the aid of which their presence is made known.

When the physician is appealed to, to pronounce upon the sex of the person, often for the purpose of reinstating an individual in his true civil rank, who has had attributed to him a sex that was not his own, he should follow from point to point the line of conduct which has already been clearly indicated by Marc: 1, to observe at length, and under several aspects, the tastes, the habits, and the moral constitution of the individual; 2, after an inspection of the entire surface of the body, to establish the sex, the characters of which seem to predominate; 3, to examine with the greatest care the external organs of generation (penis, scrotum, urethra, testicles); to sound all the openings, in order to know the extent and direction and the character of the vices of conformation, which conceal the true sex; 4, to be assured, finally, if there has ever been established an issue of catamenial blood; for this fact alone is sufficient to unveil the predominance of the attributes of the woman.

Sometimes the true sex remains unknown throughout the entire life; as in the case of Maria Arsans, who died at the age of eighty, who was a reputed woman and was married as such; but the autopsy showed in her some signs of virility. Sometimes an accidental circumstance, such as the descent of the testicles, or their strangulation in the ring, awakens the attention.

One of the most affecting and dramatic observations, is that of the poor unfortunate, who lived in a convent boarding-school, almost to the age of twenty-two years, and who, by a series of multiple circumstances, saw his own civil rank changed by a decree of the civil tribunal of Rochelle.* The history of this individual, whose name was Alexina B——, has been written by himself, and shows what terrible consequences can come from an error committed in the inscription in the civil state. This person, the prey of a miserable existence, committed suicide, in Paris, in 1868; the autopsy showed very evidently that he was a man; the body had all the exterior appearances of a man, the hair system was very well developed, the penis existed, and, moreover, there were testicles.

In order to avoid similar errors, it is especially essential to consult the physicians who have attended upon the births; "and not to be content with the declarations

* Chesnet. Annales d'hyg. et de méd. légale, 1868.

of the parents, or the persons who witnessed the accouchment, but to ascertain for one's self the sex of the infant."

Sometimes there are circumstances under which it will be very difficult to pronounce immediately; "in these cases," said M. Legrand du Saulle, "cannot one postpone the decision, as in a consultation of the guard of the seals, in 1811; and will not this postponement be preferable to a hasty affirmation, founded upon hypotheses, and analogies, and which can entail many disastrous consequences?"

One other medico-legal question should engage our attention, the solution of which, we are forced to admit, is not easy; we allude to the capability of the hermaphrodites for procreation and their fitness for marriage. Take, for example, the case of Alexina B——, in whom the penis was of small size; after the rectification of her civil rank had been obtained, could Alexina then marry? Was there really impotence? Yes; according to M. Legrand du Saulle; "because, though the act of copulation could be well accomplished, fecundation is really impossible. It remains evident that Alexina B—— is an unclassed being, incapable of reproduction, and consecrated to an eternal celibacy." It seems to me, however, that Alexina B—— presents all the attributes of the male sex, and that some day, when there will be adopted in France the singular practice of certain American surgeons, it will be no longer admissible to say that fecundation by such a subject is in reality impossible.

For the medical jurist, we will admit only two classes of hermaphrodites: in the first, much the more numerous, will be the hermaphrodites whose sex is recognizable; the most frequently they will be individuals of the male sex, who will be able to marry (the impotence not being absolute), or of the female sex who cannot marry, their sexuality not being sufficiently distinct.

In the second class, will be those individuals whose sex cannot be defined. These are the persons whom Is.

Geoffroy Saint-Hilaire designates under the name of negative, neuter hermaphrodites ; the sex of these is indeterminable ; arrested in their development, their analogue is found only in the embryo. Or rather, there may be an equal association of the two apparatuses. It is of this class of hermaphrodites, that Geoffroy Saint-Hilaire regretted "that the law admitted of only two great classes of individuals, upon whom it imposed different obligations and accorded to them different privileges."

Among these hermaphrodites, the aptitude of generation does not exist, the sexes being so imperfectly constructed, and they can neither fecundate nor conceive. Concerning the marriage, it ought to be declared null, not on account of error in the person, but because of the identity of sex of the two espoused.

In *résumé*, the arrest of development of the sexual organs gives origin, finally, to a medico-legal question of identity; this question will be solved generally, after a minute examination of the parts, being based upon the facts furnished by anatomy, physiology and embryology. Further, the greatest frequency of the vicious conformation of the male, will enable us to conclude, usually, that we have to do with individuals of the male sex.

(To be concluded.)

